# Tailored Self-Management App to Support Older Adults With Cancer and Multimorbidity: Development and Usability Testing

**DOI:** 10.2196/53163

**Published:** 2024-05-08

**Authors:** Sang-Wha Sien, Francis Kyerepagr Kobekyaa, Martine Puts, Leanne Currie, Margaret Tompson, Penelope Hedges, Joanna McGrenere, Caroline Mariano, Kristen R Haase

**Affiliations:** 1 Department of Computer Science University of British Columbia Vancouver, BC Canada; 2 Lawrence S Bloomberg Faculty of Nursing University of Toronto Toronto, ON Canada; 3 School of Nursing University of British Columbia Vancouver, BC Canada; 4 Saskatchewan Center for Patient Oriented Research Saskatoon, SK Canada; 5 BC Cancer Vancouver, BC Canada

**Keywords:** cancer, aging, self-management, usability testing, design thinking, design, oncology, develop, development, usability, gerontology, geriatric, geriatrics, older adult, older adults, elder, elderly, older person, older people, ageing, mHealth, mobile health, app, apps, application, applications, symptom, symptoms, comorbidity, comorbidities, comorbid, multimorbidity, multimorbidities, co-design

## Abstract

**Background:**

Globally, cancer predominates in adults aged older than 60 years, and 70% of older adults have ≥1 chronic condition. Cancer self-management interventions can improve symptom management and confidence, but few interventions target the complex needs of older adults with cancer and multimorbidity. Despite growing evidence of digital health tools in cancer care, there is a paucity of theoretically grounded digital self-management supports for older adults. Many apps for older adults have not been co-designed with older adults to ensure that they are tailored to their specific needs, which would increase usability and uptake.

**Objective:**

We aim to report on the user evaluations of a self- and symptom-management app to support older adults living with cancer and multimorbidity.

**Methods:**

This study used Grey’s self-management framework, a design thinking approach, and involved older adults with lived experiences of cancer to design a medium-fidelity app prototype. Older adults with cancer or caregivers were recruited through community organizations or support groups to participate in co-designing or evaluations of the app. Data from interviews were iteratively integrated into the design process and analyzed using descriptive statistics and thematic analyses.

**Results:**

In total, 15 older adults and 3 caregivers (n=18) participated in this study: 10 participated (8 older adults and 2 caregivers) in the design of the low-fidelity prototype, and 10 evaluated (9 older adults and 1 caregiver) the medium-fidelity prototype (2 older adults participated in both phases). Participants emphasized the importance of tracking functions to make sense of information across physical symptoms and psychosocial aspects; a clear display; and the organization of notes and reminders to communicate with care providers. Participants also emphasized the importance of medication initiation or cessation reminders to mitigate concerns related to polypharmacy.

**Conclusions:**

This app has the potential to support the complex health care needs of older adults with cancer, creating a “home base” for symptom management and support. The findings from this study will position the researchers to conduct feasibility testing and real-world implementation.

## Introduction

Cancer morbidity and mortality increase with age for most cancers [1,2]. With the rapidly aging population, the number of older adults (≥65 years) with cancer is estimated to double globally by 2035 [1] and triple in those aged ≥80 years in the next decades [3]. In Canada, 2 in every 5 older adults aged ≥70 years are diagnosed with cancer, accounting for 28.2% of all deaths [4]. Approximately 70% of older adults with cancer have pre-existing illnesses that occur with aging [5]. Having multiple conditions alongside cancer can lower one’s functional and cognitive status, increase the likelihood of treatment complications, and negatively impact health outcomes [1,3,5].

Given the possible deleterious side effects of cancer-related treatments, older adults and their families require self-management support during the cancer care trajectory [6,7]. Self-management refers to the ability to manage treatment effects and psychosocial changes arising as a result of illness [7]. Within the context of cancer, self-management refers to one’s ability to manage the effects of diagnosis and treatment [7]. Self-management encompasses the capacity to manage the symptoms, treatment, physical and psychosocial consequences, and lifestyle changes inherent in living with cancer [6]. Self-management support programs are often tailored to the needs and abilities of patients and their relatives [6] and comprise several core skills and responsibilities, including problem-solving, resource use, partnering with health care providers, decision-making, self-tailoring, and action planning [7]. These core skills help people with cancer and their caregivers to actively manage the illness and treatment effects, thereby reducing the effects on daily functioning and improving health [7]. For those with multimorbidity, there is more to manage, and engaging in self-management may be more challenging. Interventions that support this complexity are needed.

Digital health tools present opportunities for self-management support for older adults and caregivers [8]. A recent study by Leigh and colleagues [9] found that 68% of older adults aged ≥60 years owned and used smartphones and were interested in using mobile health apps to self-manage heart failure conditions. Mobile health apps have features that can address older adults’ needs and expectations, contributing to enhanced cancer self-management [10-12]. Cancer apps designed for older adults have been reported to enable better communication [13], the potential for patient-reported outcome collection [14], and the feasibility of electronic rapid frailty screening [15-17]. If self- and symptom-management support for older adults with cancer is tailored to the usability and capability preferences of older adults, they can optimize the management of cancer symptoms [7,8]. However, a limited number of mobile health interventions target older adults with cancer and other conditions to support self- and symptom management [10].

In our prior work with older adults, they described both the complex work of cancer self-management and a dearth of supports to manage the complex interplay of their cancer diagnosis with other illnesses [18,19]. Therefore, the purpose of this study was to address this critical gap, by reporting on the user evaluations of the design of a self-management app to support older adults living with cancer and multimorbidity. In this paper, we report on the process and outcomes of this iterative co-design process.

## Methods

### Study Design

**Figure 1:**
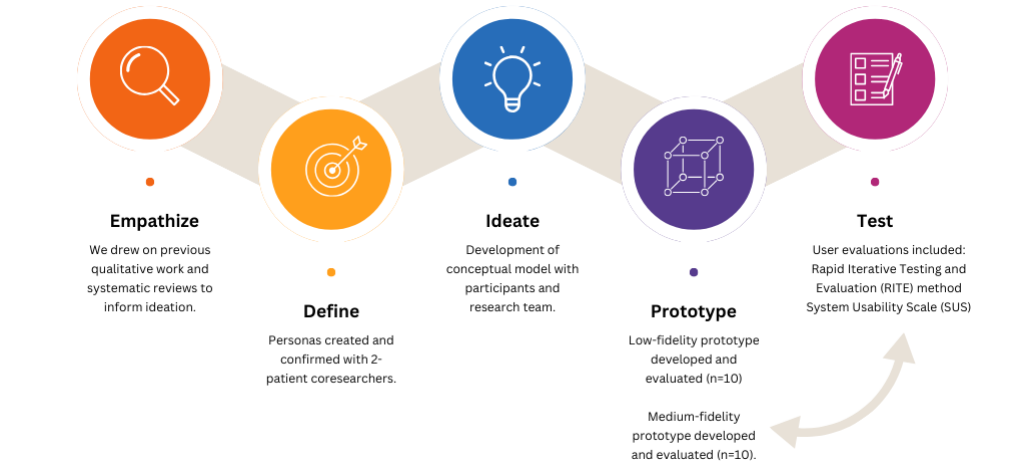
Overview of methods. Note: empathize, define, ideate, prototype, and test are the 5 stages of design thinking. RITE: Rapid Iterative Testing and Evaluation; SUS: System Usability Scale.

We used the Design Thinking model [20,21], involving a user-centered approach, with engagement from patient partners as coresearchers. A Design Thinking model involves iterative rounds of developing empathy for users, defining functional and usability requirement priorities, and ideating collective perspectives to produce a final prototype [20]. We gathered participant feedback in multiple waves to ensure that the design was user-validated at every step. Figure 1 shows an overview of our methods.

### Ethical Considerations

This study underwent ethical review by the harmonized research ethics review board at the University of British Columbia (BREB H21-03052). All participants completed an informed consent process and participated voluntarily. All personal data has been anonymized. All participants were provided an honorarium in the form of a gift card of their choice of $40 per design session.

### Recruitment and Participants

Data collection for this study took place between 2021 and 2023 in British Columbia, Canada. We used purposive sampling to recruit older adults with cancer and caregivers. The inclusion criteria for the older adults were aged ≥ 65 years; experiencing or had previously experienced cancer; had received cancer treatment within the previous year; and had at least 1 chronic illness in addition to the cancer diagnosis that required the use of medication or treatment. They also needed to have access and be able to use an internet-connected device, such as a tablet, phone, or laptop. Participants were recruited from community centers, community meetings, support groups, and a registry of patients who had participated in prior research. Caregivers were people who identified as individuals who cared for or supported an older adult during their cancer diagnosis or treatment.

We collected demographic and medical information, health literacy (using the eHealth literacy scale [eHEALS] [22]), and fitness or frailty (using the Vulnerable Elders Survey [23]) scores on all participants, over the telephone, or in person.

We recruited a total of 18 participants comprising 15 older adults and 3 caregivers. In total, 2 older adults participated in evaluating both the low-fidelity and medium-fidelity prototypes to determine whether they perceived any improvements in the design. Thus, a total of 10 participants (8 older adults and 2 caregivers) contributed to the design of the low-fidelity prototype and 10 participants (9 older adults and 1 caregiver) evaluated the medium-fidelity prototype.

Of the 18 participants, 12 (67%) of them were women and 6 (33%) were men. The ages ranged from 40 to 88 years (only age ranges were collected; half [n=9, 50%] of the participants were between 70 and 75 years old). Most were currently married or partnered (n=7, 39%) and college or university educated (n=18, 100%), and they experienced diverse cancers. When asked, they all reported feeling comfortable using the internet.

### Defining and Ideating

Using findings from previous work [18] that encapsulated the empathizing stage, we proceeded to define and ideate concepts that could help ground the prototyping phase. We broadly defined this study’s problem statement as supporting older adults’ self-management of their health to improve their quality of life. Using this definition as a prompt, as well as personas to promote empathy with the end users, the research team conducted a brainwriting exercise [24] with the target group to obtain a preliminary understanding of the key tasks that the app should support. A brainwriting exercise is an idea-generation method in human-computer interaction designed to brainstorm and generate what might be a good idea for systems design [24]. From this exercise, we shortlisted tasks that could promote users’ self-management skills according to Grey’s revised self-management theory [25] (Textbox 1). Grey’s revised self-management theory proposes that self-management for both the individual and family should be characterized as interacting with and upon a variety of proximal and distal outcomes [25]. We also highlighted tasks that promoted a more holistic and subjective understanding of users’ health, as those types of tasks could help users think about their quality of life more explicitly.

**Brainwriting outcomes mapped to Grey’s self-management theory domains: tasks and subtasks with descriptions.**

**Make daily health reports**

- Report symptoms: a daily report of the user’s symptoms, compared to the day before.
- Report events: a daily report of events that can impact the user’s symptoms, whether they are physical (taking a walk), emotional (visiting friends), or miscellaneous (weather).
- Report the day: a daily report of the day that can contextualize symptoms and events.
- Report questions and notes: a daily report of any questions or notes they may have for their health care provider.
- Report emoji: a daily report of an emoji that best represents the user’s day. Emojis are effective in comprehension and utility when understanding health reports [26].

**Learn weekly health trends**

- Read brief weekly summaries: a textual summary of the trends of a previous week.
- View visualizations of weekly data: a graphical summary that is equivalent to the textual summary. Informatics have been shown to support holistic wellness by helping older adults with decision-making and identifying trends [27].

**Schedule reminders**

- Schedule reminders to be notified of important times (medications, health care visits, and other events) [28].

**Share information**

- Email trends and daily reports to health care providers and caregivers, or download them for printing.

**Figure 2:**
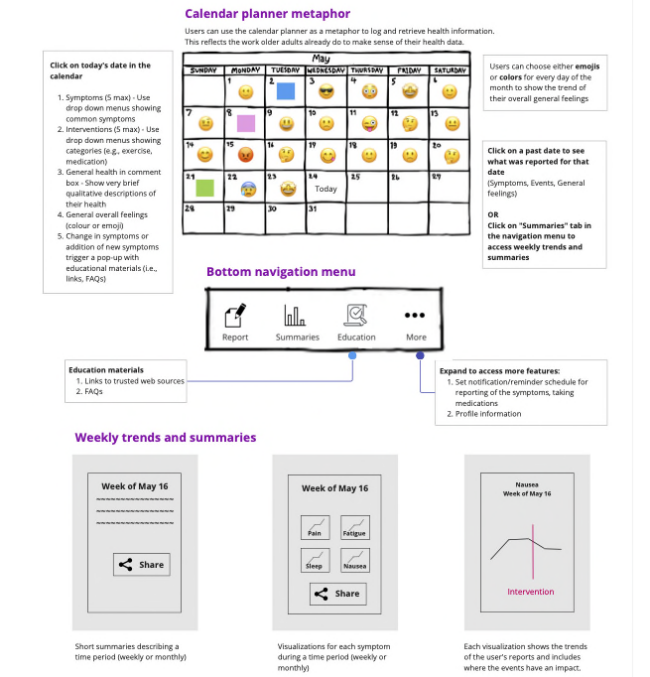
Conceptual model.

Following the brainwriting exercises, we developed a conceptual model [24] to hypothesize, at a high level, how all the tasks should fit together (Figure 2). We leveraged the metaphor of a calendar planner that older adults frequently use to record and keep a journal of their health as a starting point for our model. To validate the usefulness of the model, and to ensure we were on the right track before prototyping, we evaluated the model with 2 participants (P1 and P2) through informal interviews held on Zoom (Zoom Video Communications, Qumu Corporation). As the model was still highly conceptual, their feedback was generally well received, but with the caveat that their positive responses may change once they saw a working prototype.

### Development of Prototypes

We first acquainted ourselves with the basic human-computer interaction recommendations for older adults in interface design: minimal new concepts, plain language, unambiguous icons, accessible user interface, larger font and buttons, and consistent visual cues [29]. We also considered how to split information into smaller and more logical steps for more actionable tasks with less cognitive load [30]. This is especially important, as older adults often require more time to learn new computer skills, make more errors, and (generally) need more assistance than younger people [31,32].

We developed a low-fidelity prototype of a minimum viable product using Axure (Axure Software Solutions) and Sketch (Bohemian Coding Company) as our prototyping tools [33,34]. To rapidly assess where the app could be improved (and because we started this work in 2021 at a time when there were still limitations around social gatherings), we remotely evaluated the prototype on Zoom with 8 different participants. Participants showed how they would complete tasks on a browser and verbalized their thoughts, primarily to validate the usefulness of features and assess their high-level usability. We used the Rapid Iterative Testing and Evaluation (RITE) method, which is effective and efficient in identifying and fixing problems [35]. The main problem we identified was that the low-fidelity prototype needed to give more instructions and use plainer language. We also validated whether the visualizations would be easy to interpret, by having participants evaluate several versions of them to assess understandability: those who described having prior training to read graphs (6/8, 75%) highly preferred the visualizations, whereas the others preferred the textual summaries. This suggested that it would benefit users to implement both versions (text and graphs) in the app. Overall, the prototype was well received by all 8 participants.

Next, we proceeded with the development of the medium-fidelity prototype using Figma (Figma, Inc) as our prototyping tool [36] (Figure S1 in Multimedia Appendix 1). We also adopted the name *Mantra*, which draws on the terms “Managing Cancer” and “Comorbidity in Older Adults.” For the medium-fidelity prototype, we focused on the higher fidelity of both aesthetics and interaction compared to the low-fidelity version. The information architecture was determined by the high-level tasks, with more important task flows (ie, daily reports and weekly summaries) explicitly shown in the bottom navigation. Embedded in these 2 task flows was the ability to support information sharing. Scheduling of reminders was placed in the “More” menu (Figure S2 in Multimedia Appendix 1). The “More” menu shows the extent of design capabilities and contains unimplemented tasks to be considered for future iterations.

The task for making daily reports was designed to flow like a web-based questionnaire, with the subtasks taking up steps 1-5 (Figure S2 in Multimedia Appendix 1, shows a symptom report). To alleviate the burden of making daily reports, each step could be skipped and the system autosaved progress so that the user could continue where they had left off. The task for learning weekly health trends was designed so that the textual summary and the visualization for the same week were on the same page but in different tabs to show that they were equivalent (Figure S2 in Multimedia Appendix 1).

### Evaluations

We designed the evaluation study to be conducted both remotely and in person, as in-person evaluation may have been too physically demanding for some participants. However, we do acknowledge that the validity of the evaluation may be compromised, as the remote participants were interacting with the prototype through a browser and not on a smartphone, which has different usability concerns. We recruited 6 older adults for remote evaluations and 4 older adults for in-person evaluations of the medium-fidelity prototype.

We had three goals for the evaluation: (1) to understand whether the participants could complete the tasks (assessed through task completion rates); (2) to understand the usability of the prototype (assessed through the System Usability Scale [SUS] [37]); and (3) to understand how the app could be integrated into participants’ existing health management practices (assessed through qualitative interviews after the evaluation of the prototype; see Multimedia Appendix 2 for sample interview questions). The data collected were both qualitative and quantitative. We audio recorded and transcribed all evaluation interviews, which on average lasted 45 (SD 3.0) minutes. Thematic analysis was completed by one of the lead authors with support from the first and final authors [38,39]. We followed the 6-stage approach, which included familiarization, generating initial codes, and searching for themes. The remaining steps related to the naming and thematic structure were refined through an iterative process with weekly meetings between the 2 first authors and the senior author.

## Results

### Demographic Characteristics of Participants

In total, 15 older adults and 3 caregivers (n=18) participated in this study: 10 participated (8 older adults and 2 caregivers) in the design of the low-fidelity prototype, and 10 evaluated (9 older adults and 1 caregiver) the medium-fidelity prototype (2 older adults participated in both phases). Participants emphasized the importance of tracking functions to make sense of information across physical symptoms and psychosocial aspects symptoms; a clear display; and the organization of notes and reminders to communicate with care providers. The majority of participants were women (8/10, 80%), lived alone (6/10, 60%), and lived at home (10/10, 100%; see Table 1). Only 1 participant was considered frail according to the Vulnerable Elders Survey–13. Most (8/10, 80%) had adequate eHealth literacy according to the eHEALS (mean scores 30.6, SD 9.0), with scores ranging from 8 to 40.

**Table 1:** Sociodemographic information for all participants: older adults (n=15) and caregivers (n=3).

Characteristics		Participants (N=18), n (%)
**Age group (y)**		
	<70	4 (22)
	70-75	9 (50)
	76-80	4 (22)
	81-85	0 (0)
	>86	1 (6)
**Gender**		
	Men	6 (33)
	Women	12 (67)
**First language**		
	English	18 (100)
**Housing situation**		
	At home (house, condo, or apartment)	18 (100)
**Living situation**		
	Alone	9 (50)
	Spouse	8 (44)
	Other	1 (6)
**Marital status**		
	Married or living common law	7 (39)
	Widow or widower	3 (17)
	Separated or divorced	5 (28)
	Single (never married)	2 (11)
	Other	1 (5)
**Education level**		
	In total, 13 years and more (some or completed college or university)	18 (100)
**Type of cancer treatment (could select more than 1 type; n=15)^a,b^**		
	Surgery	13 (87)
	Radiation	6 (40)
	Chemotherapy, targeted therapy, or immunotherapy	10 (67)
	Hormone therapy	5 (33)
	Other	1 (7)
**Treatment intent as reported by patient**		
	Curative	14 (93)
	Palliative	2 (13)
**Current comorbidities**		
	Asthma, emphysema, chronic bronchitis, or COPD^c^	4 (22)
	Arthritis or rheumatism	12 (67)
	Diabetes	1 (6)
	Digestive problems (ulcer, colitis, and gallbladder disease)	5 (28)
	Heart trouble (angina, congestive heart failure, or coronary artery disease)	1 (6)
	Depression or anxiety	2 (11)
	Other	13 (72)

^a^Data collected only for older adults, not for caretakers; thus, 15 participants were included.

^b^Participants could select more than 1 option.

^c^COPD: chronic obstructive pulmonary disease.

### Task Completion and Usability

In terms of task completion, we found that 90% (9/10) of participants who evaluated the medium-fidelity prototype were able to complete all of the tasks as scored and presented in Multimedia Appendix 3. However, 1 participant (P18) experienced initial difficulty reading the text on the screen and would not have been able to complete the “Report Symptom” feature if not for the hints we provided. Nevertheless, this high completion rate indicates that the app was sufficiently designed for older adults in our target population to accomplish the key tasks.

We also assessed the general usability of the app, by administering the SUS, and found that participants evaluated the system as very usable, with an average of 87 which is described as the “best imaginable” according to Bangor and colleagues [37]. Table 2 shows the individual scores for each participant, broken down by question. Furthermore, according to Sauro and Lewis [40], the scale can be defined by 2 dimensions, that is, learnability (questions 4 and 10) and usability (all other questions). As seen in Table 2, only P18 struggled with learnability while all others perceived the app to be easy to learn. In sum, the high SUS score from the user evaluations, reflecting high user satisfaction and usability, shows that this app has great potential to assist older adults in their self-management activities.

**Table 2:** System Usability Scale responses^a^.

Participant	Q1^b^	Q2	Q3	Q4	Q5	Q6	Q7	Q8	Q9	Q10	Score
P3	5	1	4	1	4	2	5	1	5	1	92.5
P9	5	1	5	1	5	1	5	1	5	1	100
P11	4	2	4	2	4	2	4	1	5	2	80
P12	5	1	5	2	5	1	5	1	4	1	95
P13	4	1	5	1	5	1	4	1	4	2	90
P14	5	1	5	1	4	1	5	1	5	1	97.5
P15	5	1	5	1	4	1	5	1	5	1	97.5
P16	3	2	4	2	4	3	4	2	4	2	70
P17	4	1	3	1	5	1	5	1	5	1	92.5
P18	3	3	4	5	3	2	5	2	4	5	55

^a^Table explanation: there are 10 questions in total, ranging from strongly disagree (1 point) to strongly agree (5 points). The tone of the questions (see Multimedia Appendix 3) switches from positive (odd questions) to negative (even questions). The score for each person is calculated as follows: X = sum of the points for all odd-numbered questions – 5; Y = 25 – sum of the points for all even-numbered questions; individual SUS score = (X + Y) × 2.5. An average score of 85-100 is the best imaginable, while 52-85 is still considered excellent [37]. All participants evaluated the system as having high usability, except P18 who strongly agreed with the need for support from a technical person or additional knowledge to comfortably use the app.

^b^Q: question.

### Qualitative Findings

#### Overview

Based on our thematic analysis, we constructed 3 global themes from the data, related to both the nature of the app and its value in supporting self-management. These themes were organized and labeled as follows: (1) app conceptual model matches users’ mental model, (2) value and usefulness for self-management, and (3) confusing icons and buttons. These themes are discussed further below.

#### App Conceptual Model Matches Users’ Mental Model

##### Overview

In our user testing, participants found the app interfaces easy to navigate with straightforward steps to complete tasks. They also found the text summaries and data visualizations to be visually engaging with clear health-related feedback, which contributed to the ease of use of the app. In light of these user experiences, the following subthemes were constructed from the data, related to the app structure, its usability, and its ease of use: (1) transferability; (2) intuitiveness and ease of navigation; (3) clear data visualization; and (4) simplified and comprehensive summary reports.

##### Transferability

In our user testing, participants found the app to have a familiar interface, with similar features to other popular health apps that they had used or were using, which reinforced a sense of transferability. For example, 1 participant stated: “I would say that the labels were very clear to me which are similar to some of the apps I’ve used” (P15). Participants also described the app prompts as predictable with simple steps to complete tasks and found the app’s phrases and concepts to be familiar and understandable. Overall, the words and concepts used in the app followed real-world conventions which made the information appear in a logical order for participants. One of the participants said: “It was very predictable and understandable using it the first time” (P12).

##### Intuitiveness and Ease of Navigation

The app user interface offered a sharp, constant, and uncluttered background, which enhanced readability. Participants felt that the steps and buttons needed to complete the tasks were easy to navigate, which motivated them to use the app. They appreciated the simplicity of the app features which contributed to its intuitiveness. One participant commented: “I think even an elementary student could probably use this app quite easily” (P12). Furthermore, the simple interface and the easy task flow from the home page to completion, shaped user experiences as reflected here: “It was easy to use...the app is simple...but these other apps have a lot and it’s always difficult” (P14).

For some participants, the app features felt orderly and streamlined, increasing accessibility even for users with color sensitivity. The legibility and high-contrast colors of the interface alongside the large font size were perceived as being visually engaging and readable. Some participants suggested an adjustable font size should be embedded in the app to allow customization: “Everything looks clear except that the fonts needed to be adjusted big enough. I was straining to see some things” (P9).

Overall, participants found the app easy to use regardless of their level of experience with technology and cancer type. Although older adults reported feeling comfortable using the app; some users required guidance to navigate certain icons on the app. Specifically, the remote participants had more difficulty navigating the prototype if they used a phone remotely than in-person participants. One participant stated: “I had to ask for help to identify icons on the phone” (P11). Another said: “You know, I could go through it [the app] but I needed some help” (P9).

##### Clear Data Visualization

Participants’ most desired accurate unambiguous feedback with simple and clear data visualizations. Participants perceived the clarity and accuracy of the data visualizations as visually appealing, engaging, and readable.

While acknowledging color contrast in the charts and graphs, participants described how texts and labels highlighted important information. Participants reported that they could easily understand the visual elements, facilitating the effective abstraction of actionable insights from the data visuals. For instance, 1 participant stated: “The texts are fine with me...I can understand the data from the graphs” (P11). However, 1 participant highlighted their difficulty understanding the meanings of the different colors included in the charts. As a remedy, they suggested 1 color stream to ensure consistency. They also suggested that emojis should be used in place of graphs since emojis are considered a universal language for most users. Additionally, the text or data presented in the graphs might be difficult to understand and interpret by some non-English users of the app. As presented to participants, the app allowed for only 1 representative emoji per day. However, users suggested combining many emojis that best represent their emotions and feelings for the day. For example, users could combine 2 or more emojis to illustrate their emotions since they could have different experiences within a day—positive, neutral, and negative emotions: “I prefer more emojis, in fact a combination of them...because they tell more story about my emotions” (P9). Participants noted clear data visualization and accuracy of information displayed and affirmed the relevance of having readable and understandable trends of their health data tracked and presented to them in graphs covering either a month or week period.

##### Simplified and Comprehensive Summary Reports

Participants observed that the clear and comprehensive nature of the health data summaries helped to make connections between day-to-day emotions and changes over time. Some users noted the easy, actionable steps of reporting symptoms and feelings that were directly linked to the feedback. Participants found that displaying multiple types of data on 1 page was more intuitive and comprehensive and helped them make sense of their overall health:

Usually, what I see when I look at my blood reports [in leukaemia] is, I click on a report and I see a single trend line [in another app]. Now, with this app, I can see all different types of reports on one page. That’s good. It keeps it together. Because I can see that it’s helping me make connections of all the data.

P15

In comparing this app with other apps, participants noted that this app tends to present an integrated analysis of their health, emotions, and present health state, which made it more interesting to track and review. For example, 1 older adult stated:

The Apple Health app was the only one that I have used. And that tends to be reflecting sort of like a simplified quantitative analysis, not qualitative, not including emotions and things. And I think that would be an interesting thing to record and then to review. The correlation between my actual situation of cancer and how I felt about it at the time.

P15

Participants appreciated summary reports, although some older adults expressed a preference for monthly summary reports rather than weekly reports. They argued that cancer is a long-term condition that requires a summary of the symptoms over a longer period. Further, 1 participant said: “I would like a monthly report summary...the other apps I do it monthly...I will go over and look at the trend lines over the months. This would fit into that whole world that I'm a part of and have been now since 2015” (P3).

#### Value and Usefulness for Self-Management

##### Overview

Participants described how the app was valuable and useful in supporting them to manage their health, in the presence of a cancer diagnosis. Participants felt that the various dimensions of the app could support self-management as captured and explained in the following subthemes: (1) integration into existing self-management routines; (2) app design linked to user personal health needs; (3) recording and sharing of health information; and (4) digital health calendar.

##### Integration Into Existing Self-Management Routines

Our user testing demonstrated that users were ready and willing to use the app for self-management and felt the app could help them meet their health needs. App features such as the daily symptom report—which required users to report symptoms daily at a time convenient to them—were perceived as a normal daily activity that could easily be done without hassle. Participants found the app’s notifications feature a useful way to engage them and remind them to report their feelings, adhere to treatments, and honor appointments with their clinicians. For example, a participant observed that the notifications could easily be synchronized with other daily tasks:

I could see myself spending probably half an hour a day inputting information and then reviewing the results. [With another app], every morning, I wake up and check what my sleep score was and what my resting heart rate was. And when my resting heart rate goes up, it tells me that I'm not behaving myself, or stressed. So, it's just kind of a check-in with myself almost to make sure that I'm, you know, kind of following the health path that I want to be on. Again, the reminder notification is great for reporting my emotions.

P15

To encourage consistent and continuous engagement of the app in daily self-management, the app was designed to prompt users to input their health report daily.

Participants expressed that reporting their symptoms and interventions daily in the app would not impact other life activities or routines and they never found tracking in the app troubling or burdensome. Further, 1 participant said:

For me it would be because I don't have many other obligations. I'm retired. And my wife and I live at home. We don't travel much. Not at all really. So, I mean to us, you know, this is the sort of thing that I do every day. I'm on the computer doing stuff like this

P11

However, some participants felt there might be user resistance during the initial stage of the app implementation particularly for those who are not technologically savvy and may also not have a family member to guide them to navigate the app. Further, 1 participant speculated about possible resistance: “I think there's going to be some resistance to adapt for those who are not good at the computer. It's doable, but it's going to be difficult for them” (P3). Overall, participants expressed high intentions of integrating the app into their daily routines for self-management.

##### Design Linked to User Personal Health Needs

Participants reported that the app design supported their individual health needs—symptom-monitoring, tracking, and self-managing. While the app was perceived to keep users motivated, they also felt it was designed with user needs at the forefront. Further, 1 participant, who had an adult son with autism who needed to track his diet, felt the app could be useful not only in monitoring their own treatment-related symptoms, but also for noncancer patients: “Excellent, this app could align well to my health needs and also probably work well with my son too” (P3). Older adult users could in real time monitor their unique cancer symptoms, track medication adherence, and receive reminders for appointments and other relevant health updates. Further, 1 participant stated:

Well, I mean I wanted to set some goals in terms of, you know, trying to fit exercise in, and it was a way for me to challenge myself, I guess, the features that I do use are here [on this app] to receive notifications and track my health.

P9

##### Recording and Sharing of Health Information

Participants reported the value of the app’s capability to record and track information for later sharing with their clinicians. This act of keeping track of information in the form of note-taking was perceived as a better alternative to electronically record, share, and communicate users’ health issues with their health care providers than other analogue strategies. Further, 1 older adult said:

It’s a better way to organize notes and trends and see about trends. Just as a communication tool...maybe a better communication tool to use to talk to the different healthcare providers that we communicate with...rather than recording in so many places.

P13

Some participants observed that the app features mimic what older adults usually do in keeping track of events by documenting on a piece of paper their daily feelings, thoughts, and other related health issues. Some participants felt that better symptom tracking helped to gain control over symptoms and improve general well-being. Further, 1 participant stated:

So what this does is that you know. So, this feature imitates older adults, what they do with their own, like say journal or calendar or anything that they write things on. They keep track of their questions or note what they’re feeling.

P12

Older adults repeatedly voiced their willingness to use the app because it offered them the ability to share their health information with their clinicians during appointment visits. Most participants felt that clinicians could deliver more tailored care when provided with additional information during consultations.

##### Digital Health Calendar

All necessary details regarding patients’ symptom-tracking reports, treatments, and cancer education, including schedules and reminders or alerts are diarized for future reference. Participants reported how app features particularly the “reporting” and “summaries” features provide a safe place to store more secure information in the app for reference. Further, 1 participant said:

It’s like an electronic diary...a good calendar to write and keep track of notes about their feelings...So you have that instant data that you can look at where you've been and where you're going.

P14

In addition to storing information related to a person’s cancer, the app also makes it easier for the user to edit and update information with new tasks and reschedule appointments. According to participants, the app guides users to know their current state of health and to predict their future health by simply referring to the calendar. For instance, 1 older adult stated: “I mean, one of the things you can do is to keep information here [in app]...you can schedule new appointments with your doctor. It's like I could see it having lots of benefits” (P11).

Other participants emphasized the value and usefulness of the app for older adults in keeping their health records. Participants argued that most older adults are forgetful as a consequence of aging, and this app could be a great tool to help them remember their changing health. Further, 1 participant stated:

I mean, one of the things is as we age, if your memory is having trouble with remembering things, that probably helps...it would help that because then you could go back and when was I really feeling so bad and you know? That’s great to know.

P9

For some participants, this app could also help in tracking different types of treatment and associated side effects, especially for users starting new treatments. Participants also emphasized the value of this tool early in the treatment process, given the capability to track changes over time and remind users of appointments to and schedules. A participant stated:

Well, what I do is I have to go through and scroll every appointment. But where this is useful for me is when, especially when I'm starting a new treatment that I've never done before. I want to track the side effects and I want to know what the trigger points are on it...the notification reminders, it’s like a reminder of things that you have to experience. Those kinds of elements that I want to be able to look at and see how that changed over six months.

P12

##### Confusing Icons and Buttons

The last theme relates to opportunities to improve the app based on evaluations. Despite the intuitiveness and ease of navigation of the prototype, some participants found the icons and buttons confusing while others could not understand some features and information displayed. These attributes impeded usability. Further, 1 participant noted:

Consider seeking medical care. Is this with my GP? Is this with the oncologist? Do I call for medical care for fatigue? Community health news, what does this button mean? I also wonder what that recommendation is?

P3

Another participant expressed their dissatisfaction with not being able to find suitable emojis to express their emotions and asked: “I don't even see happy on the list here. Where can I find happy emoji? It's rather lots of other things here” (P9). While this participant could not find suitable emojis to express their emotions, others too found the buttons too hard to tap causing frustration as they engaged with the app prototype. A participant stated:

...because I’m not fluent with how the technology always is depicted. I tend to tap, tap, and click, click, click fast. It’s hard...It draws, but has been crazy and often I freeze something because I’ve tried to make it all go too quickly right and I’ve not given the computer time to catch what I’m doing.

P13

Participants also found some icons not matching their current state. For example, participants who are used to seeing a little pencil icon indicating “write something more” were confused when it was rather meant for “edit.” A participant stated:

I'm accustomed to seeing...the little pencil with respect to writing something more. So I think I would have assumed I should write something about nausea as opposed to edit my answer...And it’s possible that people don’t realize [it].

P3

## Discussion

### Principal Findings

In this paper, we present the findings of an iterative co-design and evaluation study of a cancer self-management app prototype designed specifically for the needs of older adults living with cancer and multimorbidity. Our key finding was that creating a space for this population to track and interpret data related to their health, in a way that made sense to them, would support their self-management needs. The app we designed built on the concept of the calendar, a routine activity that many older adults already participate in. By drawing on this common routine we developed an app that is both useful and acceptable and was not considered burdensome. This work is novel as it acknowledges the complex health states that this population experiences and aims to address their challenges through an app that was co-designed with them, for them. Our approach was user-centered, with a research team co-led by older adults with cancer experiences and comorbidities.

### Limitations

The main limitations of this study relate to the sampling. First, most of the participants were not on active treatment at the time of study participation. Future studies of the app should study usability for those with a new diagnosis using the app for the first time. Second, most participants spoke English, making usability generally easier. Third, all participants were college or university educated. Future studies should include participants with secondary education or lower to gather more broader and diverse perspectives across all levels of education and experience for the design. Finally, many of the participants regularly used technology and smartphones—however, 2 scored low on the eHEALS measure of health literacy. Nevertheless, we observe growing rates of smartphone use among older adults [41,42] and note that views that older adults do not use technology are ageist and dated. We also acknowledge that the validity of the evaluation may be compromised, as the remote participants were interacting with the prototype through a browser and not on a smartphone, which has different usability concerns. Finally, we only included participants who had their own devices, meaning that they (may) have better technical skills than those who do not own devices, and that may overestimate the usability.

### Comparison With Prior Work

This study underscores older adults’ interest in proactively managing their own cancer diagnosis and other existing conditions. While older adults’ interest in self-management has been reported in other studies [5,43], our study highlights the functionalities that support this work. Given that many older adults are managing distressing symptoms as a result of cancer and its treatments, alongside existing chronic illnesses, the impetus to mobilize self-management supports is critical [44]. Our app in its current state is designed for all older adults with cancer and other illnesses to self-manage their conditions, but it is most useful and valuable for older adults who have just been diagnosed with cancer.

Through our iterative co-design process, several noteworthy features were emphasized by older adults involved in this study. One of the features most valued by participants was the ability to facilitate meaningful connections across all aspects of health. A recent scoping review by Wilson and colleagues [45] found that apps that do not support older adults to make connections across functions were a key barrier to meaningful engagement with apps. The app developed in this study provides direction and guidance on how to report symptoms, events, and daily emotional states and provides an easy display to integrate these various inputs and share insights with health care providers. Unlike existing mobile apps with several views on multiple interfaces [46-48], this app provides a single view of all symptoms, events, and emotions consolidated on 1 page which aims to improve usability, efficiency, and satisfaction among users. The design also allows an effective presentation of daily reports in a comprehensive and easy-to-understand manner. Additionally, the app’s design exhibits flexibility, allowing older adults—in collaboration with their health clinicians and caregivers—to prioritize specific symptoms (or variables) of focus. These features are in sharp contrast to existing apps and websites used by older adults to track health information, which often have a narrow scope, concentrating solely on particular cancers or symptoms [27,49-52]. Health apps with narrow scopes may hamper a comprehensive understanding of health and quality of life from a multidimensional perspective [27].

While other studies detail older adults reporting difficulties with limited functionalities and comprehension of visual health data [45,53], participants in this study emphasized a preference for both text and visual summaries to help them make sense of patterns in the data. Older adults also appreciated and valued different modes to view data trends. For example, notes and textual summaries served as an alternative means of conveying information for those who struggled with graphical representations. This has been reported in other studies both within cancer [54] and elsewhere [55]. For example, in a recent pilot randomized controlled trial by Lally and colleagues [54], older adults expressed a preference for information leaflets and text notes to describe the trends of their health. Our study reiterates those findings and also highlights the possibility of text summaries becoming “a voice” for older adults during consultations with their clinicians. Text summaries allow for better and more streamlined conversations with clinicians, thereby promoting shared decision-making [56,57]. It is important to note that the preferences and recommendations of our participants informed revisions of the app features and functionalities in the prototype iterations, and the current state of the app reflects the needs of its potential users. Overall, the app developed in this study represents an acceptable and usable app that is adaptable to the unique needs of older adults with cancer in monitoring changes in their health.

### Conclusions

Our self-management app prototype has both content and face validity among older adults with cancer and comorbidities. At this stage, the app requires further refinements and testing to understand its efficacy and to gauge its acceptability and implementation potential within the cancer care system in Canada and beyond.

## Appendix

Multimedia Appendix 1 Medium-fidelity prototype.

Multimedia Appendix 2 Interview sample questions.

Multimedia Appendix 3 System Usability Scale questions (rated between strongly agree to strongly disagree).

## Abbreviations

eHEALS: eHealth literacy scale
RITE: Rapid Iterative Testing and Evaluation
SUS: System Usability Scale
