# Supplementary material for: Tailored Self-Management App to Support Older Adults With Cancer and Multimorbidity: Development and Usability Testing
Source: JMIR Aging. 2024 May 8;7:e53163. doi: 10.2196/53163 (PMC11112470; doi:10.2196/53163)
Supplement: Multimedia Appendix 1 [file aging_v7i1e53163_app1.docx]

**Multimedia Appendix 1.**

Figure S1. Landing pages and additional screen caps for app demonstration purposes.

| 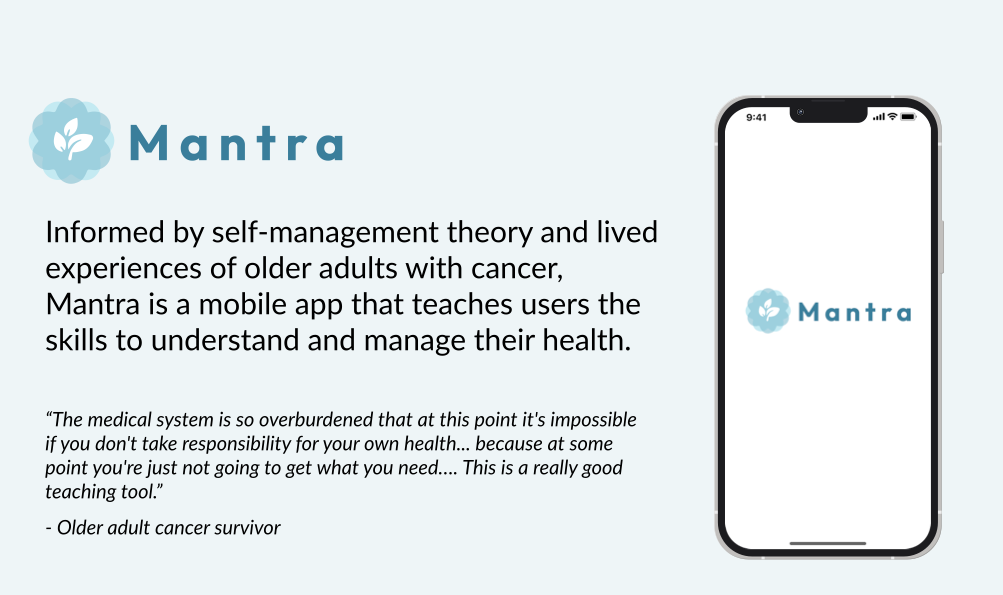  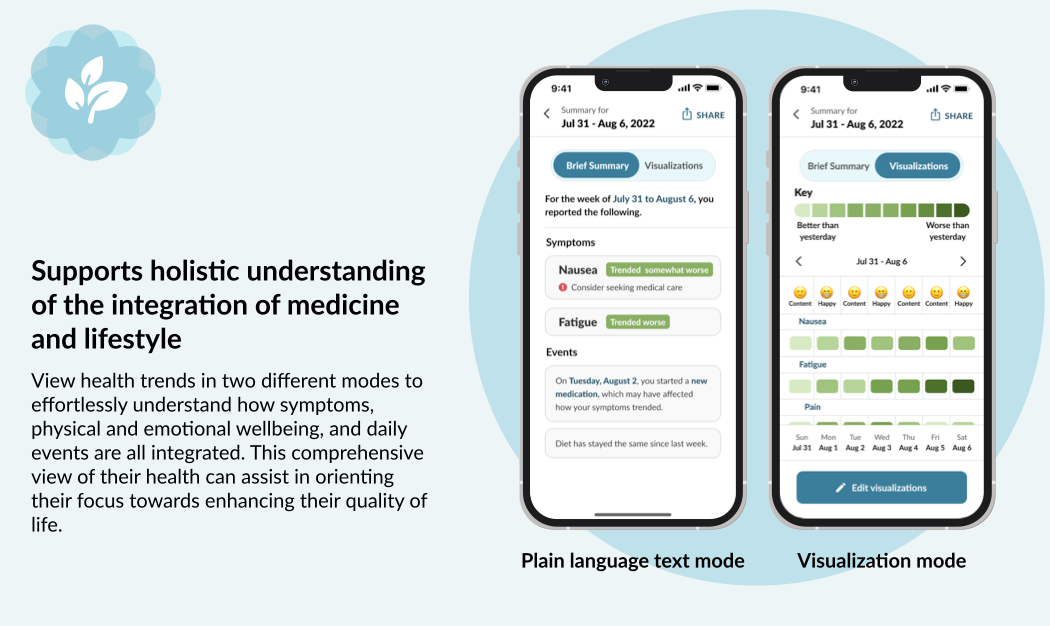  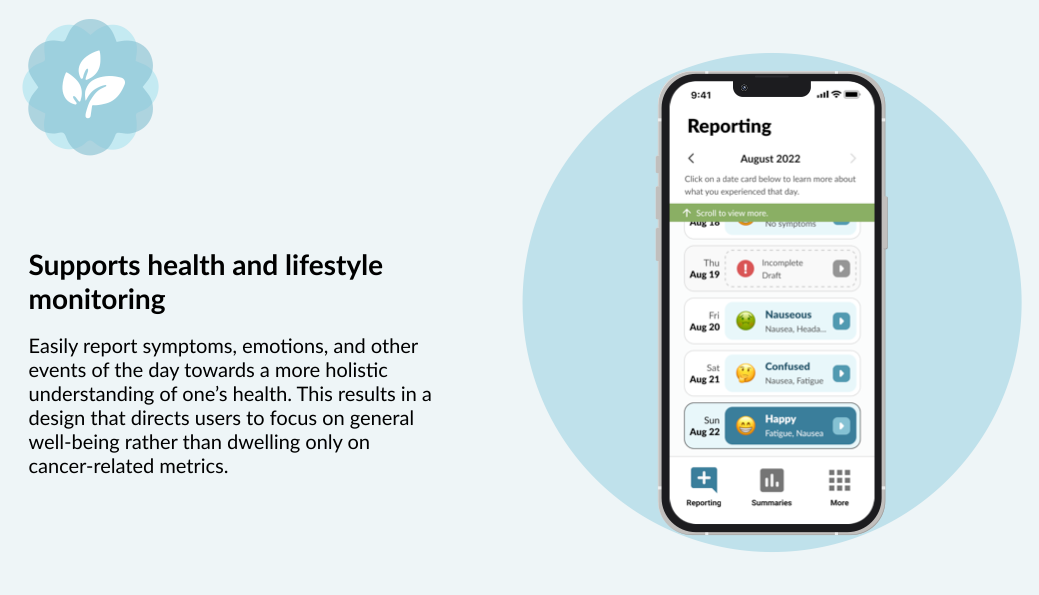  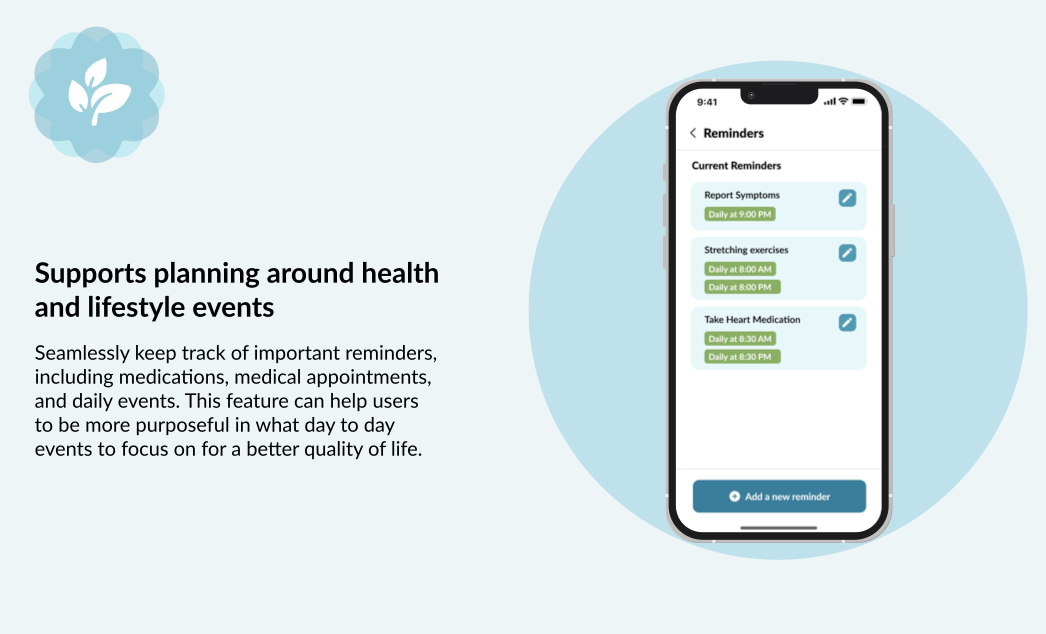 |
| --- |

Figure S2. More menus.

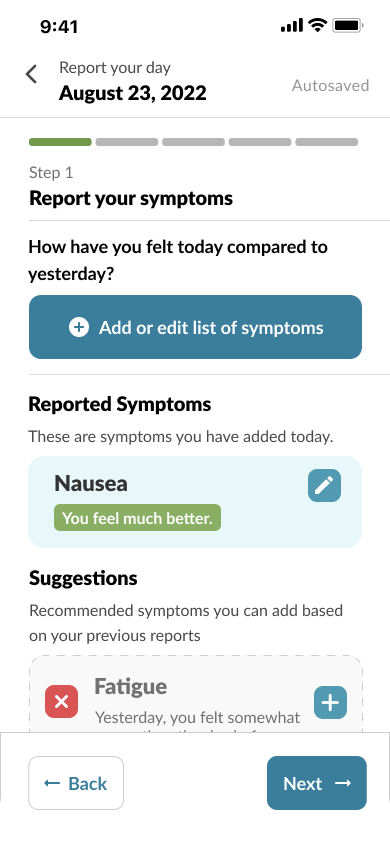


| 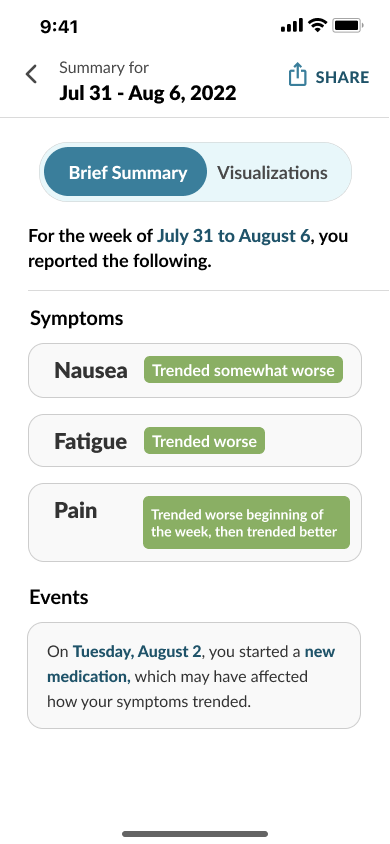 | 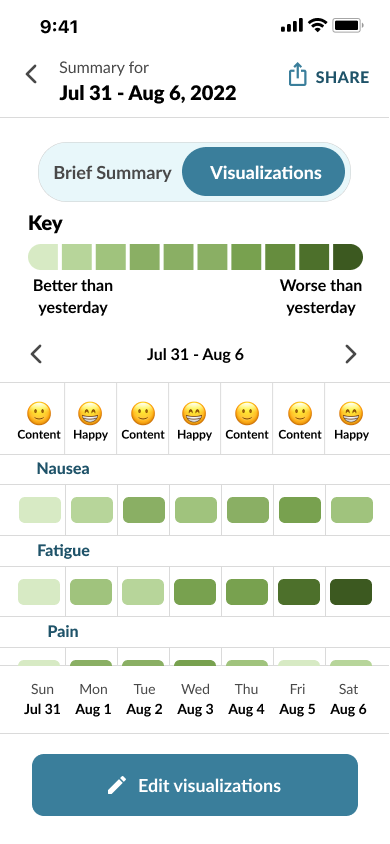 |
| --- | --- |
