# Supplementary material for: Tailored Self-Management App to Support Older Adults With Cancer and Multimorbidity: Development and Usability Testing
Source: JMIR Aging. 2024 May 8;7:e53163. doi: 10.2196/53163 (PMC11112470; doi:10.2196/53163)
Supplement: Multimedia Appendix 2 [file aging_v7i1e53163_app2.docx]

**Multimedia Appendix 2.** Interview sample questions.

**General user experience**

1. Can you describe your overall experience in using ***the app***?
2. Do you feel ***the app*** can be integrated in your regular care with your healthcare team?
3. Is there anything specific you like/dislike about this system overall?
4. If given the option, would you use this outside of the study?
5. One of the key features centers around making connections across everything you’ve reported (for example, the symptoms, events, and emotions). How do you feel about this feature? Would it be valuable for you, or not?

**Interactions** a

1. Do you find the features and functions displayed clear to read and understand?
2. When you just started using ***the app***, did you have any difficulty understanding the information displayed?
3. Would you like to see a different way to DISPLAY INFO / CONTENT/TRACKING?
4. Is there anything specific you like/dislike about the presentation of ***the app***?
5. Depending on the final design, suggest prompts: visualization, display, entering responses, etc.

**Perceived value**

1. Do you find it useful to use ***the app***?

**Accuracy vs accessibility**

1. Was the information, layout, etc. clear and accessible?

**Acceptance of *the app***

1. Would you use the self-management recommendations and tracking tools?
2. Is there anything specific you like/dislike about ***the app***?
3. Prompt discussion about: form factor, accessibility, trust in digital info, etc.
4. There is quite a bit of work regarding using this app. It’s expected that a user would need to make a report every day. How do you feel about this? Would this be feasible in your current lifestyle?
5. How does the prototype fit in to your current ways of managing your health?
