# Supplementary material for: Tailored Self-Management App to Support Older Adults With Cancer and Multimorbidity: Development and Usability Testing
Source: JMIR Aging. 2024 May 8;7:e53163. doi: 10.2196/53163 (PMC11112470; doi:10.2196/53163)
Supplement: Multimedia Appendix 3 [file aging_v7i1e53163_app3.docx]

## Multimedia Appendix 3. System Usability Scale questions (rated between strongly agree to strongly disagree).

1. I think that I would like to use this website frequently.
2. I found the website unnecessarily complex.
3. I thought the website was easy to use.
4. I think that I would need the support of a technical person to be able to use this website.
5. I found the various functions in this website were well integrated.
6. I thought there was too much inconsistency in this website.
7. I would imagine that most people would learn to use this website very quickly.
8. I found the website very cumbersome to use.
9. I felt very confident using the website.
10. I needed to learn a lot of things before I could get going with this system.
